# Mono‐ and Co‐Culture Biofilms of *Candida auris* or *Candida albicans* With *Staphylococcus aureus* Regulate Cell Viability and Pro‐Inflammatory Cytokine Expression Differently in Oral Cancer Cell Lines

**DOI:** 10.1111/jop.70043

**Published:** 2025-08-20

**Authors:** Wan NurHazirah Wan Ahmad Kamil, Munirah Mokhtar, Madia Baizura Baharom, H. M. H. N. Bandara, Mohammad S. Alrashdan, Nicola Cirillo, Mohd Hafiz Arzmi

**Affiliations:** ^1^ Department of Fundamental Dental and Medical Sciences, Kulliyyah of Dentistry International Islamic University Malaysia Kuantan Pahang Malaysia; ^2^ Cluster of Cancer Research Initiative IIUM (COCRII) International Islamic University Malaysia Kuantan Pahang Malaysia; ^3^ Centre of Oral Maxillofacial Diagnostic Medicine and Studies, Faculty of Dentistry Universiti Teknologi MARA (UiTM) Sungai Buloh Selangor Malaysia; ^4^ Department of Medical Diagnostic, Faculty of Health Sciences Universiti Selangor Shah Alam Malaysia; ^5^ Department of Basic Medical Sciences, Kulliyyah of Medicine International Islamic University Malaysia Kuantan Pahang Malaysia; ^6^ Bristol Dental School University of Bristol Bristol UK; ^7^ Department of Oral and Craniofacial Health Sciences, College of Dental Medicine University of Sharjah Sharjah UAE; ^8^ Department of Oral Medicine and Oral Surgery, Faculty of Dentistry Jordan University of Science and Technology Irbid Jordan; ^9^ Melbourne Dental School The University of Melbourne Melbourne Victoria Australia

**Keywords:** biofilms, *Candida albicans*, *Candida auris*, co‐culture, mono‐culture, pro‐inflammatory cytokines, *Staphylococcus aureus*

## Abstract

**Background:**

*Candida* species and *Staphylococcus aureus* are nosocomial pathogens associated with immunocompromised individuals, especially oral cancer patients. This study elucidates the effects of mono‐ and co‐culture biofilms of *Candida albicans* , *Candida auris* and *S. aureus* on cell viability and pro‐inflammatory cytokine expression in healthy epithelial cells (hTERT TIGKs) and oral cancer (ORL‐48) cell lines.

**Methods:**

Mono‐ and co‐culture biofilms of *C. albicans* , *C. auris* and *S. aureus* , developed using static biofilm for 72 h, were collected and filter sterilized (biofilm filtrate). Test cell growth medium (TCGM) was prepared for hTERT TIGKs and ORL‐48 by mixing 20% (v/v) biofilm filtrate with 80% serum‐free media. The cells were seeded in 96‐well plates, and TCGM was added as treatment. The unstimulated media (UM), made of 100% serum‐free media, served as control. After 24 h, cell viability was assessed using CCK‐8. Interleukin (IL)‐6 and IL‐8 expression was analysed using enzyme‐linked immunosorbent assay (ELISA).

**Results:**

*S. aureus* consistently decreased cell vitality in co‐culture as compared to mono‐culture, *C. albicans* and *C. auris* in both cell lines. Co‐culture of *C. auris* and *S. aureus* in cancer cells demonstrated significantly higher IL‐6 and IL‐8 expression than *C. albicans* (*p* < 0.05). Furthermore, co‐culturing *C. auris* or *C. albicans* with *S. aureus* increases pro‐inflammatory IL‐8 expression in oral cancer cell lines compared to mono‐cultures of respective *Candida*.

**Conclusion:**

Co‐culturing *C. auris* or *C. albicans* with *S. aureus* reduced cell viability and increased pro‐inflammatory IL‐8 expression in oral cancer cell lines, suggesting that interkingdom interaction may regulate oral carcinogenesis.

## 1 Introduction

Oral squamous cell carcinoma (OSCC) is the most pervasive form of head and neck cancer and is characterised by a high mortality rate, a tendency for recurrence and a potential for metastasis [1]. The main risk factors for OSCC include tobacco use, alcohol consumption, betel quid chewing and microbial infections regarded as an emerging risk factor [2]. However, the role of oral microbial infections in the development of oral cancer and its biological mechanisms remains poorly understood. The potential carcinogenic mechanisms influenced by oral infections include cytokine production and the induction and exacerbation of chronic inflammation [2]. Microbial infections also regulate cellular viability, migration and apoptosis by disrupting the cell cycle and activating anti‐apoptotic signalling pathways [2, 3].

The interactions between *Candida auris* or *Candida albicans* and *Staphylococcus aureus* contribute to the development of nosocomial infections. They can also promote chronic inflammation, modulate immune responses and induce a resistant and pro‐migratory phenotype, which, in turn, contributes to tumour progression. Furthermore, these pathogens can form biofilms that modify the production of inflammatory cytokine molecules, such as interleukin (IL)‐6 and IL‐8, in both healthy and cancer cell lines [4].

Although several studies have reported interactions between *C. albicans* and *S. aureus* in oral cell models [5], the effects of intra‐ and interkingdom biofilms of the emerging nosocomial fungal pathogen, *C. auris*, on cell viability and pro‐inflammatory cytokines expression from oral cancer cell lines remain unknown. Therefore, this study aimed to determine the effect of mono‐ and co‐culture biofilms of *Candida* spp. and *S. aureus* in normal (hTERT TIGKs) and oral cancer (ORL‐48) cell lines, with the hypothesis that interkingdom biofilms modulate cell viability and pro‐inflammatory cytokines in oral keratinocytes.

## 2 Materials and Methods

### 2.1 Cell Lines and Culture

Normal human gingival keratinocytes immortalised with hTERT (hTERT TIGKs) were purchased from the American Type Culture Collection (ATCC) (CRL‐3397, Manassas, VA).

The oral cancer cell line ORL‐48, derived from malignant keratinocytes of the gingiva of an oral cancer patient, was obtained from Cancer Research Malaysia, Subang Jaya Medical Centre (CRM, Selangor, Malaysia). Specific culture conditions are reported in the Supporting Information S1.

### 2.2 Preparation of Test Cell Growth Medium (TCGM)

The mono‐ and co‐culture biofilms were developed previously, prior to the collection of TCGM as described by Wan Ahmad Kamil et al. [6]. Details for the production of biofilms are reported in the Supporting Information S1. After 72 h of biofilm formation, the biofilm filtrate was collected and filter‐sterilised using a 0.22 μM Millex Millipore syringe filter (MERCK, Germany). To prepare TCGM for hTERT TIGKs and ORL‐48, 20% (v/v) biofilm filtrate was mixed with dermal cell basal medium (PCS‐200‐030; ATCC, Manassas, VA) and DMEM/F12 (Thermo Fisher Scientific, USA), respectively.

### 2.3 Cell Viability Assay Using Cell Counting Kit‐8 (CCK‐8)

hTERT TIGKs and ORL‐48 cells were incubated with TCGM or unstimulated media, and the cell viability assay was undertaken as detailed in the Supporting Information S1. Absorbance was measured at 450 nm using an Infinite 200 Pro microplate reader (TECAN, Switzerland).

### 2.4 Pro‐Inflammatory Cytokines Expression

ELISA assay was conducted to assess the expression of pro‐inflammatory cytokines. In brief, the samples are prepared using the same method as for the CCK‐8 assay. Following the procedure, the conditioned medium was collected for ELISA and transferred into an ELISA plate to measure the concentrations of IL‐6 and IL‐8 using an ELISA (Elabscience, USA) based on the manufacturer's instructions. The procedure is detailed in the Supporting Information S1.

### 2.5 Statistical Analysis

All data were analysed using GraphPad Prism version 10.0 and IBM SPSS Statistics for Mac, version 29.0 (IBM Corp., Armonk, NY, USA). A two‐tailed paired *t* test was used to compare co‐culture in different cell lines. A one‐way analysis of variance (ANOVA) with Dunnett's post hoc test was performed to compare cell viability and cytokine expression amongst the biofilm‐conditioned media groups. A *p* value < 0.05 was considered statistically significant.

## 3 Results

### 3.1 Effect of Biofilm on the Cell Viability of hTERT TIGKs and ORL‐48 Oral Cell Lines

The largest number of viable cells (hTERT TIGKs and ORL‐48) was observed within 24 h treatment with mono‐culture *C. auris* biofilm‐conditioned media, whereas the lowest was observed when treated with mono‐culture *S. aureus* biofilm‐conditioned media (Table S1). After 24 h treatment with co‐culture biofilm of *C. auris and S. aureus*, the cell viability of hTERT TIGKs and ORL‐48 was significantly decreased as compared to mono‐culture of both *C. auris* (*p* < 0.001) and *C. albicans* (*p* < 0.001) (Table 1).

**TABLE 1:** One‐way analysis of variance (ANOVA) with Dunnett's T3 post hoc test comparing: (a) cell viability of hTERT TIGKs and ORL‐48 amongst the biofilms conditioned media groups; (b) pro‐inflammatory cytokine interleukin‐6 (IL‐6) produced by hTERT TIGKs and ORL‐48 amongst the biofilm conditioned media groups; and (c) pro‐inflammatory cytokine interleukin‐8 (IL‐8) produced by hTERT TIGKs and ORL‐48 amongst the biofilm conditioned media groups.

					95% CI		
Cell lines	Multiple comparisons (Post hoc Dunnet T3)			Mean difference	Lower	Upper	*p*
*(a) Cell viability of hTERT TIGKs and ORL‐48 amongst the biofilms conditioned media groups* ^a^							
hTERT TIGKs	Mono‐culture *C. auris*	vs.	*C. auris* + *S. aureus* (co‐culture)	0.085	0.081	0.089	< 0.001
	Mono‐culture *C. auris*	vs.	Unstimulated media (UM)	0.126	0.122	0.130	< 0.001
	*C. auris* + *S. aureus* (co‐culture)	vs.	Unstimulated media (UM)	0.041	0.036	0.046	< 0.001
	Mono‐culture *C. albicans*	vs.	*C. albicans* + *S. aureus* (co‐culture)	0.027	0.016	0.039	< 0.001
	Mono‐culture *C. albicans*	vs.	Unstimulated media (UM)	0.087	0.077	0.097	< 0.001
	*C. albicans* + *S. aureus* (co‐culture)	vs.	Unstimulated media (UM)	0.041	0.036	0.461	< 0.001
	Mono‐culture *C. auris*	vs.	Mono‐culture *C. albicans*	0.039	0.029	0.049	< 0.001
	*C. auris* + *S. aureus* (co‐culture)	vs.	*C. albicans* + *S. aureus* (co‐culture)	0.018	0.009	0.028	< 0.001
ORL‐48	Mono‐culture *C. auris*	vs.	*C. auris* + *S. aureus* (co‐culture)	0.034	0.031	0.037	< 0.001
	Mono‐culture *C. auris*	vs.	Unstimulated media (UM)	0.082	0.079	0.085	< 0.001
	*C. auris* + *S. aureus* (co‐culture)	vs.	Unstimulated media (UM)	0.048	0.044	0.052	< 0.001
	Mono‐culture *C. albicans*	vs.	*C. albicans* + *S. aureus* (co‐culture)	−0.014	−0.036	0.007	0.372
	Mono‐culture *C. albicans*	vs.	Unstimulated media (UM)	0.023	0.004	0.042	< 0.001
	*C. albicans* + *S. aureus* (co‐culture)	vs.	Unstimulated media (UM)	0.009	−0.007	0.024	0.500
	Mono‐culture *C. auris*	vs.	Mono‐culture *C. albicans*	0.059	0.034	0.078	< 0.001
	*C. auris* + *S. aureus* (co‐culture)	vs.	*C. albicans* + *S. aureus* (co‐culture)	0.040	0.024	0.055	< 0.001
*(b) Pro‐inflammatory cytokine interleukin‐6 (IL‐6) produced by hTERT TIGKs and ORL‐48 amongst the biofilm conditioned media groups* ^b^							
hTERT TIGKs	Mono‐culture *C. auris*	vs.	*C. auris* + *S. aureus* (co‐culture)	−25.481	−25.503	−25.459	< 0.001
	Mono‐culture *C. auris*	vs.	Unstimulated media (UM)	16.491	16.383	16.600	< 0.001
	*C. auris* + *S. aureus* (co‐culture)	vs.	Unstimulated media (UM)	41.973	41.868	42.077	< 0.001
	Mono‐culture *C. albicans*	vs.	*C. albicans* + *S. aureus* (co‐culture)	58.717	58.550	58.884	< 0.001
	Mono‐culture *C. albicans*	vs.	Unstimulated media (UM)	76.136	76.019	76.253	< 0.001
	*C. albicans* + *S. aureus* (co‐culture)	vs.	Unstimulated media (UM)	17.419	17.298	17.540	< 0.001
	Mono‐culture *C. auris*	vs.	Mono‐culture *C. albicans*	−59.645	−59.668	−59.621	< 0.001
	*C. auris* + *S. aureus* (co‐culture)	vs.	*C. albicans* + *S. aureus* (co‐culture)	24.554	24.396	24.711	< 0.001
ORL‐48	Mono‐culture *C. auris*	vs.	*C. auris* + *S. aureus* (co‐culture)	0.601	0.428	0.774	0.03
	Mono‐culture *C. auris*	vs.	Unstimulated media (UM)	0.410	0.277	0.544	0.04
	*C. auris* + *S. aureus* (co‐culture)	vs.	Unstimulated media (UM)	1.011	0.875	1.147	< 0.001
	Mono‐culture *C. albicans*	vs.	*C. albicans* + *S. aureus* (co‐culture)	1.037	1.006	1.067	< 0.001
	Mono‐culture *C. albicans*	vs.	Unstimulated media (UM)	1.740	1.601	1.879	< 0.001
	*C. albicans* + *S. aureus* (co‐culture)	vs.	Unstimulated media (UM)	0.703	0.573	0.834	0.001
	Mono‐culture *C. auris*	vs.	Mono‐culture *C. albicans*	1.330	1.302	1.357	< 0.001
	*C. auris* + *S. aureus* (co‐culture)	vs.	*C. albicans* + *S. aureus* (co‐culture)	0.308	0.138	0.478	0.14
*(c) Pro‐inflammatory cytokine interleukin‐8 (IL‐8) produced by hTERT TIGKs and ORL‐48 amongst the biofilm conditioned media groups* ^a^							
hTERT TIGKs	Mono‐culture *C. auris*	vs.	*C. auris* + *S. aureus* (co‐culture)	35.227	16.718	53.736	0.014
	Mono‐culture *C. auris*	vs.	Unstimulated media (UM)	59.500	40.361	78.629	0.005
	*C. auris* + *S. aureus* (co‐culture)	vs.	Unstimulated media (UM)	24.268	21.688	26.849	< 0.001
	Mono‐culture *C. albicans*	vs.	*C. albicans* + *S. aureus* (co‐culture)	2.478	0.145	4.811	0.043
	Mono‐culture *C. albicans*	vs.	Unstimulated media (UM)	10.280	7.282	13.279	0.004
	*C. albicans* + *S. aureus* (co‐culture)	vs.	Unstimulated media (UM)	7.802	6.393	9.212	0.002
	Mono‐culture *C. auris*	vs.	Mono‐culture *C. albicans*	49.214	30.907	67.523	0.006
	*C. auris* + *S. aureus* (co‐culture)	vs.	*C. albicans* + *S. aureus* (co‐culture)	16.466	14.528	18.404	< 0.001
ORL‐48	Mono‐culture *C. auris*	vs.	*C. auris* + *S. aureus* (co‐culture)	30.165	29.622	30.707	< 0.001
	Mono‐culture *C. auris*	vs.	Unstimulated media (UM)	14.759	14.515	15.002	< 0.001
	*C. auris* + *S. aureus* (co‐culture)	vs.	Unstimulated media (UM)	44.923	44.267	45.580	< 0.001
	Mono‐culture *C. albicans*	vs.	*C. albicans* + *S. aureus* (co‐culture)	27.600	27.123	28.067	< 0.001
	Mono‐culture *C. albicans*	vs.	Unstimulated media (UM)	22.730	22.362	23.098	< 0.001
	*C. albicans* + *S. aureus* (co‐culture)	vs.	Unstimulated media (UM)	42.354	41.768	42.939	< 0.001
	Mono‐culture *C. auris*	vs.	Mono‐culture *C. albicans*	−7.971	−8.256	−7.686	< 0.001
	*C. auris* + *S. aureus* (co‐culture)	vs.	*C. albicans* + *S. aureus* (co‐culture)	2.569	2.064	3.075	< 0.001

Abbreviation: CI, confidence interval.

^a^ The data represent the mean difference from three biological replicates, with each replicate consisting of three technical replicates (*n* = 9). Data was considered significantly different when *p* < 0.05.

^b^ The data represent the mean and standard deviation (SD) from three biological replicates, with each replicate consisting of three technical replicates (*n* = 9). Data was considered significantly different when *p* < 0.05.

**FIGURE 1:**
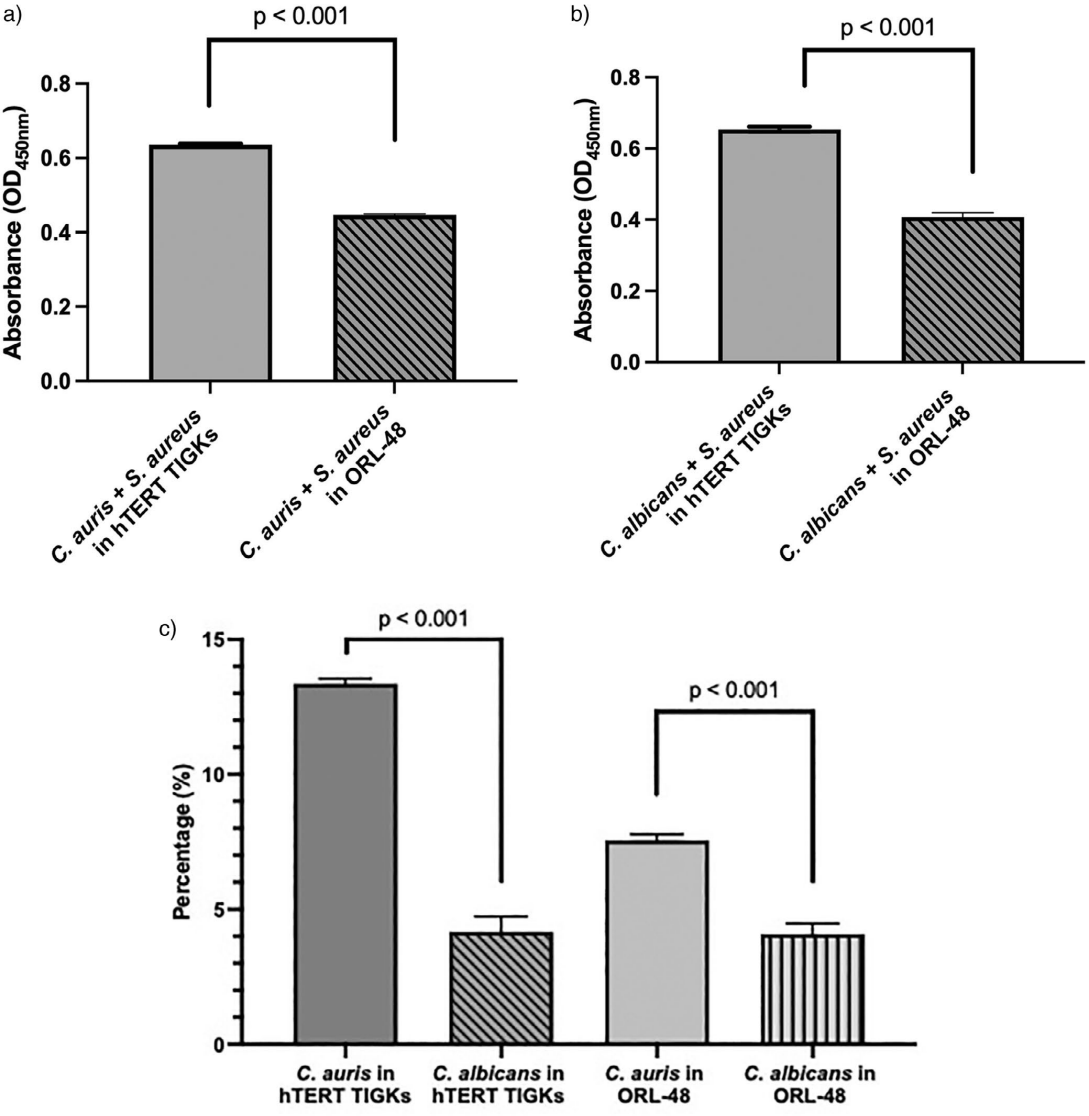
The cell viability of hTERT TIGKs and ORL‐48 based on the absorbance at OD_450 nm_ when treated with co‐culture of *Candida* with *Staphylococcus aureus* . Significant differences were observed between the co‐culture of *Candida auris* (a) or *Candida albicans* (b) with *S. aureus* in different cell lines. (c) The percentage reduction was measured by comparing biofilm‐conditioned media of *S. aureus* and *Candida* with respective mono‐culture in hTERT TIGKs and ORL‐48 cell viability. The data represent the means from three biological replicates, each consisting of three technical replicates (*n* = 9). The data were analysed using a two‐tailed paired *t* test. A significant difference in cell viability between *Candida* spp. in respective cell lines was observed (*p* < 0.05).

The cell viability of hTERT TIGKs when treated with co‐culture of *C. auris* or *C. albicans* with *S. aureus* was significantly higher than ORL‐48 cells (*p* < 0.001; Figure 1a,b). In addition, a significantly higher percentage reduction in the number of viable cells was observed when hTERT TIGKs and ORL‐48 cell lines were treated with *C. auris* co‐cultured with *S. aureus* compared to co‐culture of *C. albicans* (*p* < 0.001; Figure 1c).

### 3.2 Effect of Biofilm‐Conditioned Media on Pro‐Inflammatory Cytokine Expression in hTERT TIGKs and ORL‐48 Cell Lines

The expression of pro‐inflammatory cytokines in hTERT TIGKs and ORL‐48 cell lines varied under different biofilm culture media conditions. Overall, both mono‐cultures and co‐culture of *C. auris* or *C. albicans* with *S. aureus* significantly increased IL‐6 and IL‐8 expression in hTERT TIGKs and ORL‐48 as compared to UM (Tables S2 and S3, respectively).

**FIGURE 2:**
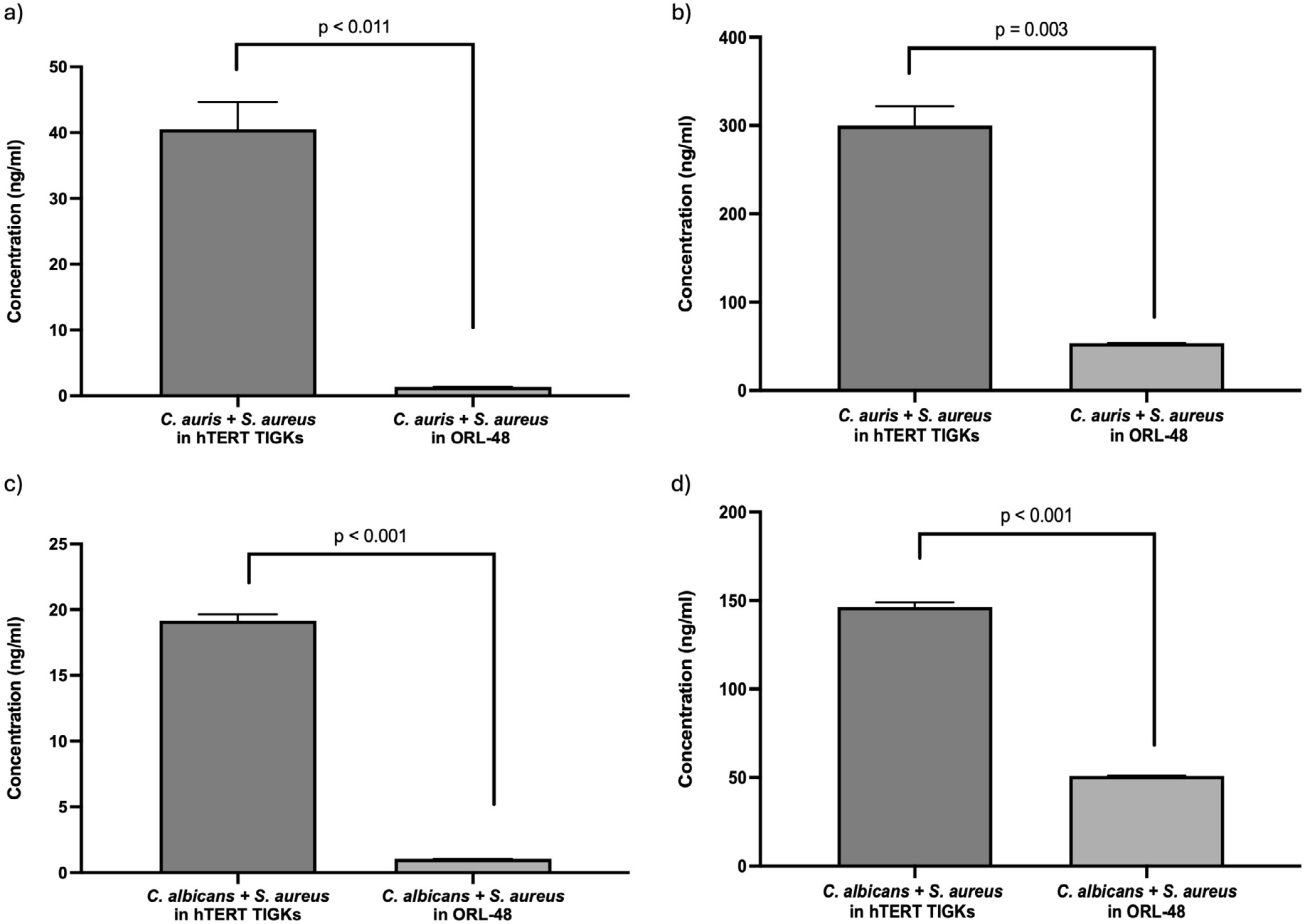
(a, c) IL‐6 and (b, d) IL‐8 cytokine expression of hTERT TIGKs and ORL‐48 based on the absorbance at OD_450 nm_ when treated with co‐culture of *Candida* with *Staphylococcus aureus* . The data were analysed using a two‐tailed paired *t* test. Significant differences were observed between the co‐culture of *Candida auris* or *Candida albicans* with *S. aureus* in different cell lines. Data were considered significantly different when (*p* < 0.05).

The expression of IL‐6 in hTERT TIGKs and ORL‐48 significantly increased when treated with co‐culture *C. auris* and *S. aureus* as compared to mono‐culture *C. auris* (*p* < 0.001; *p* = 0.03; Table 1), whereas the opposite was true when *C. albicans* was used (*p* < 0.001; *p* < 0.001; Table 1). The expression of IL‐8 in hTERT TIGKs significantly decreased during co‐culture with either *C. auris* or *C. albicans* with *S. aureus* as compared to mono‐culture of respective *Candida* spp. (Table 1). In addition, significant differences in IL‐6 and IL‐8 cytokine expression were observed between the co‐culture of *C. auris* or *C. albicans* with *S. aureus* in different cell lines (*p* < 0.05; Figure 2).

## 4 Discussion

The present study revealed mono‐ and co‐culture biofilms of *Candida* spp. and *S. aureus* promote cell growth and modulate cytokine production in oral keratinocytes in a paracrine, interkingdom and cell‐specific manner.

Mono‐culture biofilms of *C. auris* exhibited the highest growth‐stimulating capacity in both hTERT TIGKs and ORL‐48 cell lines. In contrast, treatment with co‐culture biofilms of *C. auris* or *C. albicans* and *S. aureus* significantly reduced cell viability in both cell lines compared to mono‐culture of the respective *Candida*. This suggests that *S. aureus*, which produces exotoxins such as α‐hemolysin and leukocidins, can paracrinally compromise the cell viability of hTERT TIGKs [5].

The expression of IL‐6 in hTERT TIGKs and, to a lesser extent, in ORL‐48, was increased when treated with co‐culture biofilm‐conditioned media of *C. auris* with *S. aureus* compared to mono‐culture *C. auris* . Conversely, a reduction in the expression of IL‐6 was observed in both cell lines when treated with co‐culture of *C. albicans* and *S. aureus* , compared to mono‐culture *C. albicans* . The results highlight that oral biofilms formed by nosocomial pathogenic bacteria and fungi may contribute to oral mucosal inflammation and are consistent with research indicating that co‐infections and microbial interactions can exacerbate chronic diseases and could be involved in the progression of oral inflammatory diseases and oral cancer [7, 8, 9].

The present study also showed an increase of IL‐8 expression in malignant ORL‐48 cells, but not in hTERT TIGKs, when treated with co‐culture biofilms of *C. auris* or *C. albicans* with *S. aureus* as compared to mono‐culture of the yeast. Overall, the findings are significant in oral oncology in that they demonstrate a specific increase in IL‐8 expression, which is potentially a biomarker for oral cancer [5], when coinfected by *C. auris* and *S. aureus* biofilms. As previous studies consistently reported a higher level of IL‐8 in carcinomas directly linked with tumour angiogenesis and disease severity [10], our results suggest that interkingdom interaction might increase tumour vascularity and disease progression in oral cancer patients.

In conclusion, this study demonstrates for the first time that *C. auris* biofilm creates a favourable environment for cell survival and growth and that co‐culture of *C. auris* or *C. albicans* with *S. aureus* increases pro‐inflammatory IL‐8 expression in oral cancer cell lines, but not in non‐malignant cells. Thus, interkingdom interaction may regulate oral carcinogenesis.

## Author Contributions

W.N.W.A.K.: data curation, formal analysis. M.M. and M.H.A.: methodology, project administration, resources, review and editing. M.B.B.: investigation, methodology. H.M.H.N. and M.S.A.: review and editing. W.N.W.A.K. and M.H.A.: conceptualisation, investigation, methodology, supervision, resources, visualisation. W.N.W.A.K., N.C., and M.H.A.: writing (original draft), review and editing.

## Ethics Statement

This study did not involve human participants or animal subjects, and therefore, ethical approval was not required.

## Consent

The authors have nothing to report.

## Conflicts of Interest

The authors declare no conflicts of interest.

## Peer Review

The peer review history for this article is available at https://www.webofscience.com/api/gateway/wos/peer‐review/10.1111/jop.70043.

## Supporting information


**Data S1:** Supporting Information.

**Table S1:** Descriptive analysis of cell viability in hTERT TIGKs and ORL‐48 cells based on the absorbance at OD_450nm_. when treated with biofilm‐conditioned media of mono‐and co‐culture *C. auris* or *C. albicans* with *S. aureus* .

**Table S2:** Descriptive analysis of pro‐inflammatory cytokines interleukin‐6 (IL‐6) expressed in hTERT TIGKs and ORL‐48 cell lines when treated with biofilm‐conditioned media of mono‐and co‐culture *C. auris* or *C. albicans* with *S. aureus* .

**Table S3:** Descriptive analysis of pro‐inflammatory cytokines interleukin‐8 (IL‐8) expressed in hTERT TIGKs and ORL‐48 cell lines when treated with biofilm‐conditioned media of mono‐and co‐culture *C. auris* or *C. albicans* with *S. aureus* .

## Data Availability

The data that support the findings of this study are available on request from the corresponding author. The data are not publicly available due to privacy or ethical restrictions.
