# Supplementary material for: Mono‐ and Co‐Culture Biofilms of Candida auris or Candida albicans With Staphylococcus aureus Regulate Cell Viability and Pro‐Inflammatory Cytokine Expression Differently in Oral Cancer Cell Lines
Source: J Oral Pathol Med. 2025 Aug 20;54(9):909–15. doi: 10.1111/jop.70043 (PMC12521062; doi:10.1111/jop.70043)
Supplement: Supplementary file 1 — Data S1: Supporting Information. [file JOP-54-909-s004.docx]

**Supplementary 1 (S1)**

**Materials and Methods**

***Culture conditions***

The hTERT TIGKs cell lines were grown in a complete medium consisting of 11.64 ml of Dermal Cell Basal Medium (PCS-200-030; ATCC, Manassas, VA) and 0.36 ml of Keratinocyte Growth Kit (PCS-200-400; ATCC, Manassas, VA) in a 75 cm² flask (Thermo Fisher Scientific Nunc, USA) and incubated at 37 °C, 5% CO_2_.

The ORL-48 cell were cultured in 12 ml of Dulbecco’s modified Eagle’s medium/Nutrient mixture F-12 (DMEM)/F12 media (Thermo Fisher Scientific, USA) supplemented with 10% Fetal bovine serum (FBS, Thermo Fisher Scientific, USA) and 1% antibiotics comprising 10,000 IU penicillin and 10 mg/mL streptomycin (10,000 U/mL) (Thermo Fisher Scientific, USA) and 0.5 μg ml⁻¹ hydrocortisone (Sigma–Aldrich, Dorset, UK).

The cells were cultured as a monolayer and permitted to achieve approximately 80% confluence in a humidified incubator at 37°C with 5% CO2 (Esco, Singapore), utilizing T25 and T75 flasks (Thermo Fisher Scientific Nunc, USA).

***Mono- and co-culture biofilm formation***

The yeasts (*C. albicans* (ALC2) isolated from the oral sample of an AIDS patient [17], and *C. auris* (MYA-5002; ATCC, Manassas, VA) [18], were standardised at 10^6^ cells mL^-1^. Meanwhile, *S. aureus* (25923; ATCC, Manassas, VA) [19], was standardised and 10^7^ cells mL^-1^. Then, 1 mL of suspension containing 6 x 10^6^ cells of *Candida* spp. or 6 x 10^7^ cells of *S. aureus* was pipetted into a separate well of a sterile 6-well plate (Nunc, Denmark). Then, 1 mL of brain heart infusion supplemented with yeast extract (BHIYE) (Thermo Fisher Scientific, USA) broth was added to each well. The BHIYE was prepared by dissolving 37 g of the brain heart infusion (BHI) broth (Thermo Fisher Scientific, USA) powder and adding 5 g of yeast extract in 1 L of distilled water (Thermo Fisher Scientific, USA) [20]. Then, the plate was incubated at 37 °C for 72 h to induce biofilm maturation [17,21,22]. The medium was aseptically replenished every 24 h. This same method was used for co-culture biofilms, where *C. albicans* or *C. auris* were combined with *S. aureus* in the same well of sterile 6-well plates at a similar total cell count as in the mono-culture.

The biofilm formation for mono- and co-culture of *C. albicans*, *C. auris* and *S. aureus* was validated via total CFU/mL, biomass measurement using Crystal Violet staining, and microscopy techniques as described in our previous study, confirming that these cultures formed biofilm communities.

***Cell viability assay***

hTERT TIGKs and ORL-48 cell suspensions were seeded into a 96-well plate containing 200 µL of complete medium with a seeding density of approximately 3 x 10^4^ cells, respectively. The cells were incubated at 37 °C with 5% CO_2_ until 95% confluency. The complete medium was removed upon reaching 95% confluency (approximately 24 h incubation). Then, the treatment was performed by adding the test cell growth medium to each well. Meanwhile, the unstimulated media (UM) treatment, consisting of 100% serum-free media, was pipetted into the control well.

The cells were seeded in triplicate in two different 96-well plates for each cell line. Then, the 96-well plate was incubated at 37 °C with 5% CO_2_ for 24 h. After 24 h incubation, 10 μL of CCK-8 (Med Chem Express, USA) solution was added to each well, and the plate was incubated for 2 h in the incubator. After 2 h of incubation, the plate was mixed gently in an orbital manner for 1 min to ensure a homogeneous distribution of colour, and the absorbance was measured at 450 nm using an Infinite® 200 Pro microplate reader (TECAN, Switzerland). The experiment was performed in three biological replicates.

***Cytokine expression***

The expression of IL-6 and IL-8 was assessed using the ELISA Immunoassay Kit (Elabscience®, USA), which contains cytokine-specific monoclonal antibody pre-coated ELISA plates, following the method described by Bhardwaj et al. (2020) [24]. In brief, 100 μL of conditioned medium was added to the well of the ELISA plate and incubated at 37°C for 90 min. After removing the suspension, 100 μL of Biotinylated Detection Ab working solution was pipetted into each well. The plate was incubated for 60 min at 37°C and washed thrice. Then, 100 μL of the HRP conjugate working solution was dispensed into each well, followed by a 30 min incubation period at 37°C. After washing the plate five times, 90 μL of substrate reagent was pipetted. The plate was then incubated at 37°C for 15 min.

To conclude the procedure, 50 μL of stop solution was pipetted into each well. Subsequently, a spectrophotometer was used to measure the optical density (OD) at 450 nm wavelength. The OD value is proportional to the concentrations of human IL-6 and IL-8. The minimum detectable concentrations for the ELISA are 1.56 pg/mL and 7.81 pg/mL for IL-6 and IL-8, respectively. The experiment included two biological and two technical replicates. The sample replicate count followed the ELISA protocol from Elabscience®, USA.

***Normality of distribution***

Normality of the variable distribution was assessed by examining skewness and kurtosis, with values between −2 and +2 interpreted as acceptable indicators of approximate normality. The similarity between the mean and median, along with a standard deviation that, when doubled, did not exceed the mean, further supported the assumption of normality.
