# Supplementary material for: Mono‐ and Co‐Culture Biofilms of Candida auris or Candida albicans With Staphylococcus aureus Regulate Cell Viability and Pro‐Inflammatory Cytokine Expression Differently in Oral Cancer Cell Lines
Source: J Oral Pathol Med. 2025 Aug 20;54(9):909–15. doi: 10.1111/jop.70043 (PMC12521062; doi:10.1111/jop.70043)
Supplement: Supplementary file 2 — Table S1: Descriptive analysis of cell viability in hTERT TIGKs and ORL‐48 cells based on the absorbance at OD450nm. when treated with biofilm‐conditioned media of mono‐and co‐culture C. auris or C. albicans with S. aureus . [file JOP-54-909-s001.docx]

**Supplementary Tables**

**Supplementary Table 1**

Descriptive analysis of cell viability in hTERT TIGKs and ORL-48 cells based on the absorbance at OD_450nm_. when treated with biofilm-conditioned media of mono-and co-culture *C. auris* or *C. albicans* with *S. aureus.*

| **Cell lines** | **Microorganisms** | **Absorbance at OD_450nm_**  **Mean ± SD** | **95% CI** | | **p value** | **ANOVA**  **Effect sizes^a^** |
| --- | --- | --- | --- | --- | --- | --- |
|  |  |  | **Lower** | **Upper** |  |  |
| hTERT TIGKs | *C. auris* (mono-culture) | 0.720 ± 0.001 | 0.712 | 0.721 | p < 0.001 | 0.940 |
|  | *C. auris + S. aureus* (co-culture) | 0.635 ± 0.003 | 0.633 | 0.638 |  |  |
|  | *C. albicans* (mono-culture) | 0.681 ± 0.008 | 0.675 | 0.687 |  |  |
|  | *C. albicans + S. aureus* (co-culture) | 0.654 ± 0.007 | 0.649 | 0.659 |  |  |
|  | *S. aureus* (mono-culture) | 0.514 ± 0.037 | 0.486 | 0.542 |  |  |
|  | Unstimulated media (UM) | 0.594 ± 0.003 | 0.591 | 0.597 |  |  |
| ORL-48 | *C. auris* (mono-culture) | 0.481 ± 0.001 | 0.480 | 0.482 | p < 0.001 | 0.952 |
|  | *C. auris + S. aureus* (co-culture) | 0.447 ± 0.003 | 0.445 | 0.449 |  |  |
|  | *C. albicans* (mono-culture) | 0.422 ± 0.015 | 0.411 | 0.434 |  |  |
|  | *C. albicans + S. aureus* (co-culture) | 0.408 ± 0.012 | 0.399 | 0.417 |  |  |
|  | *S. aureus* (mono-culture) | 0.383 ± 0.009 | 0.376 | 0.390 |  |  |
|  | Unstimulated media (UM) | 0.399 ± 0.002 | 0.398 | 0.401 |  |  |

The data represent the mean and standard deviation (SD) from three biological replicates, with each replicate consisting of three technical replicates (n=9). Data was considered significantly different when p < 0.05. CI represents confidence interval, and ^a^eta-squared was estimated based on the fixed effect model.
